# *Caenorhabditis elegans* glutamylating enzymes function redundantly in male mating

**DOI:** 10.1242/bio.017442

**Published:** 2016-09-15

**Authors:** Daniel G. Chawla, Ruchi V. Shah, Zachary K. Barth, Jessica D. Lee, Katherine E. Badecker, Anar Naik, Megan M. Brewster, Timothy P. Salmon, Nina Peel

**Affiliations:** Department of Biology, The College of New Jersey, Ewing, NJ 08618, USA

**Keywords:** Microtubule, Glutamylation, *C. elegans*

## Abstract

Microtubule glutamylation is an important modulator of microtubule function and has been implicated in the regulation of centriole stability, neuronal outgrowth and cilia motility. Glutamylation of the microtubules is catalyzed by a family of tubulin tyrosine ligase-like (TTLL) enzymes. Analysis of individual TTLL enzymes has led to an understanding of their specific functions, but how activities of the TTLL enzymes are coordinated to spatially and temporally regulate glutamylation remains relatively unexplored. We have undertaken an analysis of the glutamylating TTLL enzymes in *C. elegans*. We find that although all five TTLL enzymes are expressed in the embryo and adult worm, loss of individual enzymes does not perturb microtubule function in embryonic cell divisions. Moreover, normal dye-filling, osmotic avoidance and male mating behavior indicate the presence of functional amphid cilia and male-specific neurons. A *ttll-4(tm3310); ttll-11(tm4059); ttll-5(tm3360)* triple mutant, however, shows reduced male mating efficiency due to a defect in the response step, suggesting that these three enzymes function redundantly, and that glutamylation is required for proper function of the male-specific neurons.

## Abstract (precis)

**Summary:** Although mutations in individual microtubule glutamylating enzymes do not disrupt essential microtubule functions in *C. elegans*, combining mutations in three enzymes uncovers a redundant function for glutamylation in male mating.

## INTRODUCTION

Microtubules are a major component of the cellular cytoskeleton and play essential roles in intracellular transport, cell division and cilia structure. The microtubules are subject to a vast array of post-translational modifications, including acetylation, glutamylation, tyrosination, and glycylation, and this extraordinary complexity has led to the proposal that it forms a ‘tubulin code’ (Garnham et al., 2015; Verhey and Gaertig, 2007). The tubulin code, it has been suggested, differentiates subpopulations of microtubules and regulates the binding of proteins that modulate microtubule function. The expression of enzymes that post-translationally modify the microtubules and the availability of cellular effectors would therefore converge to regulate microtubule function.

One widespread post-translational modification of the microtubule is glutamylation, which involves the reversible covalent linkage of glutamic acid to a residue within the C-terminal tail of tubulin. The initial γ-linked side-branch can be elongated by stepwise addition of further glutamates, linked by regular peptide bonds, making variable length polyglutamate chains. Tubulin modification, including glutamylation, is catalyzed by members of the tubulin tyrosine ligase-like (TTLL) family of enzymes (Janke et al., 2005). Mammals have 13 predicted TTLL enzymes, of which nine possess glutamylating activity. Each TTLL enzyme shows a preference for either α- or β-tubulin as a substrate, and has primarily side-chain-initiating or side-chain-elongating activity (van Dijk et al., 2007). The diversity of glutamylating enzymes and their spatial and temporal distribution is thought to contribute to the complex patterns of glutamylation that are observed within and between tissues (Eddé et al., 1990; Kann et al., 2003; Yu et al., 2015).

Glutamylation is enriched on the microtubules of the centriole, cilia and axons, and its role is slowly being elucidated (Bobinnec et al., 1998b; Bré et al., 1994; Fouquet et al., 1996; Kann et al., 2003; Lechtreck and Geimer, 2000). In addition to functions in centriole stability and neurite outgrowth, glutamylation appears to play a major role in modulating cilia function (Bobinnec et al., 1998a; Ikegami et al., 2007). Depletion of select TTLL enzymes in *Chlamydomonas* and *Tetrahymena* revealed a requirement for glutamylation in cilia motility (Kubo et al., 2010; Suryavanshi et al., 2010). Moreover, loss of individual TTLL enzymes in mice impairs motility of ependymal cilia, airway cilia and the sperm flagellum (Bosch Grau et al., 2013; Ikegami et al., 2010; Lee et al., 2013).

Accumulating evidence suggests that glutamylation regulates microtubule behavior by modulating interactions between the microtubule and motors, or other microtubule-associated proteins. In mice, loss of TTLL1 activity led to a decrease in the affinity of kinesin 3 for the microtubule, and *in vitro* assays indicate that glutamylation increases the motility of kinesin 1 and 2 (Ikegami et al., 2007; Sirajuddin et al., 2014). Within the cilium, glutamylation controls the interaction between inner-arm dynein and microtubules of the axoneme to regulate microtubule sliding (Kubo et al., 2010; Suryavanshi et al., 2010). Glutamylation also modulates interaction of the microtubules with microtubule-severing enzymes such that hyperglutamylated microtubules form a preferential substrate for the microtubule-severing enzyme spastin (Lacroix et al., 2010). Tubulin glutamylation by the TTLL enzymes is therefore emerging as an important contributor to microtubule function.

How activities of the individual TTLL enzymes are coordinated to spatially and temporally regulate glutamylation remains elusive. Analyses of the TTLL enzymes are beginning to reveal the specific functions of individual enzymes, and a recent analysis of all TTLLs in mouse ependymal cells has given a more comprehensive picture of TTLL activities in these multiciliated cells (Bosch Grau et al., 2013); however, a global analysis of TTLL function *in vivo* is still lacking. *Caenorhabditis elegans* affords important advantages for the study of tubulin glutamylation. First, the *C. elegans* genome encodes only five glutamylating enzymes, all of which are widely expressed in the adult worm (Janke et al., 2005; Kimura et al., 2010). Second, *C. elegans* lacks glycylation, a competing microtubule modification that has complicated analyses in other organisms (Kimura et al., 2010). Although the spatial distribution of the TTLL enzymes in the adult worm has been reported (Kimura et al., 2010) a comprehensive analysis of *C. elegans* TTLL function has not been presented.

Here we report the analysis of all five *C. elegans* glutamylating enzymes, TTLL-4, -5, -9, -11, and -15. We find that although all five enzymes are expressed in both the embryo and in the adult worm, individual loss of any enzyme does not perturb the function of the centriole or cytoplasmic microtubules. Moreover both amphid cilia and male-specific neuronal function is retained. Combinatorial loss of three TTLL enzymes, however, leads to a defect in male mating efficiency, indicating that the TTLL enzymes function redundantly in the male-specific neurons.

## RESULTS

### Domain structure of the *C. elegans* TTLL proteins

**Fig. 1.**
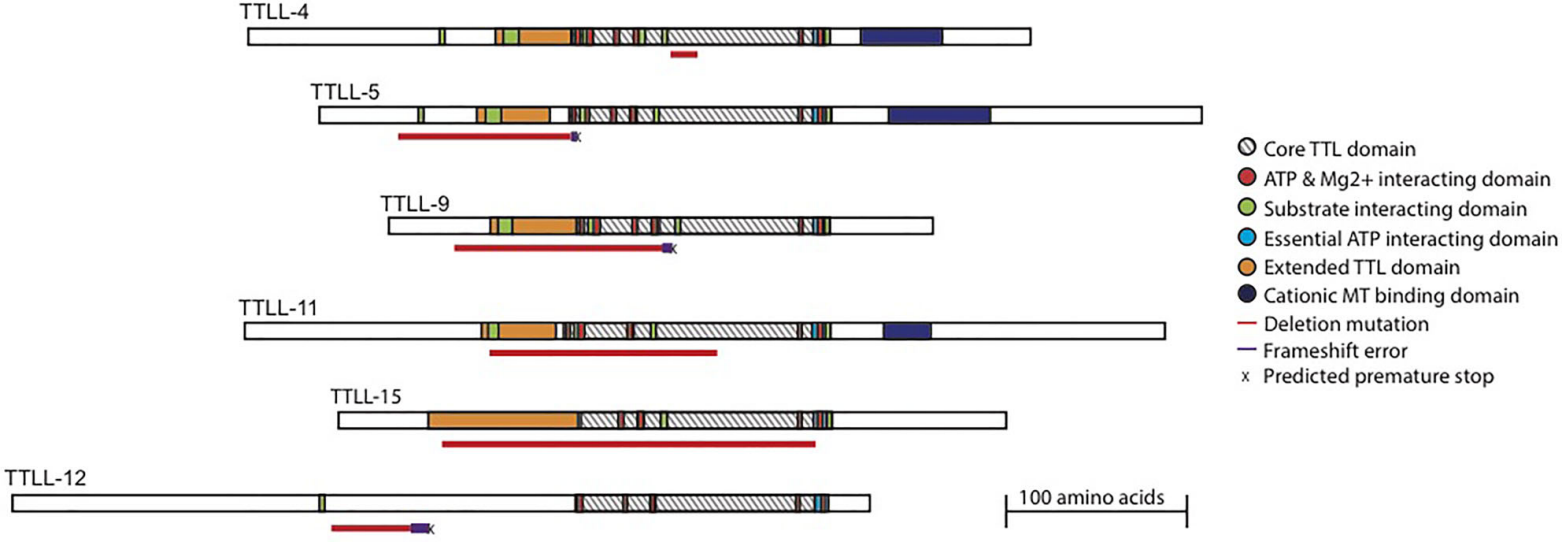
**Predicted domain structure of *C. elegans* TTLL proteins.** *C. elegans* TTLL protein sequences were annotated after alignment with mouse proteins. Deletion mutations used in this study are indicated beneath each protein in red. If a deletion is predicted to cause a frameshift it is denoted by a thicker, purple line following the deletion. Annotated sequences are available (Supplementary data).

Bioinformatics searches have revealed that the *C. elegans* genome encodes six TTLL enzymes (Janke et al., 2005). Analyses of murine glutamylases have revealed a core TTL domain containing the essential ATPase site common to all TTLL enzymes (van Dijk et al., 2007). In addition, TTLL enzymes which act as glutamylases have an extended TTL domain containing elements required for interaction with tubulin and glutamate substrates. In order to determine whether these functional elements of the TTLL enzymes are present in the *C. elegans* proteins, we aligned each *C. elegans* protein with its murine ortholog. Because TTLL-15 does not have a direct murine ortholog we aligned it with the most closely related, TTLL5. Using these alignments we were able to infer functional domains in the *C. elegans* proteins based on annotations of the murine sequences (Fig. 1 and Supplementary data). The core and extended TTL domains were conserved in *C. elegans* TTLL-4, -5, -9, -11 and -15 proteins, implying that these enzymes possess glutamylating activity. *C. elegans* TTLL-12, like its homologs that do not show glutamylating activity, lacks the extended TTL domain, suggesting that it is not a glutamylating enzyme (Brants et al., 2012; Janke et al., 2005; van Dijk et al., 2007). We have focused our analyses on the five *C. elegans* glutamylating enzymes: TTLL-4, -5, -9, -11 and -15.

Interaction of some TTLL enzymes with the microtubule substrate additionally requires a cationic microtubule binding domain (cMTBD) (Garnham et al., 2015). A cMTBD domain has been identified in monomeric mouse enzymes including TTLL4, TTLL5 and TTLL11, but is absent from mouse TTLL9, which probably uses a binding partner to interact with the microtubule (Garnham et al., 2015; Kubo et al., 2013). *C. elegans* homologs TTLL-4, -5 and -11 have cation-enriched regions, but we were unable to identify a cMTBD in either TTLL-9 or TTLL-15 (Fig. 1). This suggests that although TTLL-4, -5 and -11 can directly contact the microtubules, TTLL-9 and TTLL-15 may require additional factors to mediate this interaction.

### All five enzymes are expressed in both the adult worm and in the embryo

**Fig. 2.**
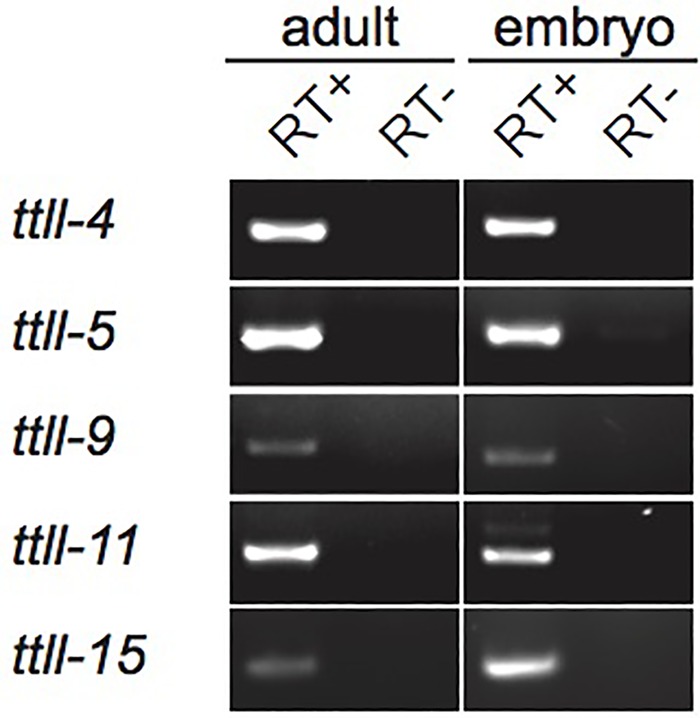
**Expression patterns of the five TTLL enzymes.** Reverse transcription PCRs were carried out on RNA extracted either from whole adult worms or from embryos. A PCR product of the appropriate size was detected in both adult and embryo (RT+). The RT− control (reverse transcriptase omitted) confirmed products did not result from DNA contamination.

Mice possess nine TTLL enzymes with glutamylating activity, however most tissues express only a subset of these enzymes (Bosch Grau et al., 2013; Janke et al., 2005; van Dijk et al., 2007). This tissue-specific expression of TTLL enzymes contributes to the establishment of complex patterns of microtubule modification, and could result in tissue-specific functions for glutamylation. Reporter constructs have previously shown cell-type-specific expression patterns for the TTLL enzymes in the adult worm (Kimura et al., 2010), however this analysis used worms carrying extrachromosomal transgene arrays, which have limited usefulness in the germline and embryo due to germline transgene silencing (Praitis et al., 2001). Therefore to test for expression of the *C. elegans* TTLL enzymes in the embryo we carried out a reverse transcription analysis on RNA extracted from embryos and adult worms. We found that transcripts of all five TTLL enzymes were present in both adult and embryonic tissues (Fig. 2). Expression of all the enzymes in both the adult and embryo led us to ask whether they have essential functions at these stages.

### Individual loss of the TTLL enzymes does not impact embryonic viability or spindle function

In order to assess the function of the individual TTLL enzymes, we obtained a deletion mutation for each (Gengyo-Ando and Mitani, 2000). We confirmed the presence of each deletion and outcrossed the strain for a minimum of six generations. We mapped each deletion on to the annotated protein sequence to determine the likely impact on protein function (Fig. 1). The *ttll-4(tm3310)* deletion allele is the smallest deletion, but removes a portion of the essential core TTL domain, including substrate interaction motifs within the active site. Deletion of an equivalent region from TTL renders it inactive (Szyk et al., 2011) and the presence of the *tm3310* mutation in TTLL-4 leads to a drastic reduction in glutamylated microtubules in sensory cilia, suggesting that it is a strong loss-of-function allele (Kimura et al., 2010). The deletions in the *ttll-5(tm3360)* and *ttll-9(tm3889)* alleles occur very early in the gene and likely lead to frameshift mutations (Fig. 1), presumably completely removing protein function. The *ttll-11(tm4059)* and *ttll-15(tm3871)* alleles are large in-frame deletions that remove the essential core TTL and extended TTL domains. All of the mutations remove essential components of the respective enzyme, resulting in loss-of-function, presumably null, alleles.

**Fig. 3.**
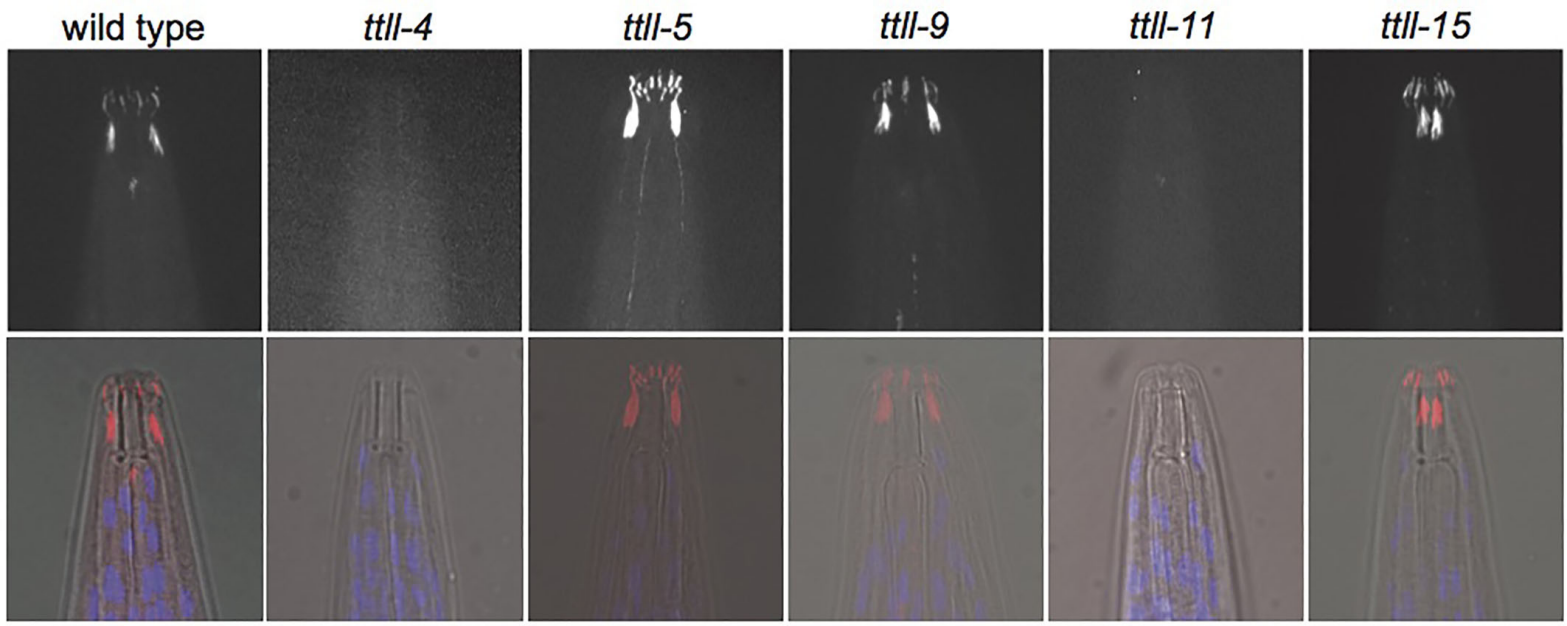
**Individual loss of some, but not all, TTLL enzymes reduces glutamylation.** Top row, adult worms of the indicated genotypes stained with anti-glutamylated tubulin (GT335) antibody. Bottom row, GT335 (red) and DNA (blue) staining overlaid on a brightfield image of each worm.

To assess whether tubulin glutamylation was impaired in the single mutants we fixed adult worms and stained with the GT335 antibody that recognizes glutamylated tubulin in the ciliated sensory neurons of the head (Bobinnec et al., 2000; Kimura et al., 2010) (Fig. 3). GT335 staining becomes undetectable in *ttll-4* and *ttll-11* mutants suggesting that these mutations severely impair enzyme function. In contrast, levels of glutamylation were comparable to wild type in *ttll-5*, *ttll-9* and *ttll-15* single mutant worms. Since together TTLL-4 and TTLL-11 provide key glutamylating activities in the amphid neurons it seems likely that redundancy is masking the effect of individual loss of *ttll-5*, *ttll-9* and *ttll-15* enzymes. Given the nature of these mutations (all delete essential domains) we considered it pertinent to further analyze the phenotypes of all the single mutants in order to infer functions for the individual enzymes.

**Fig. 4.**
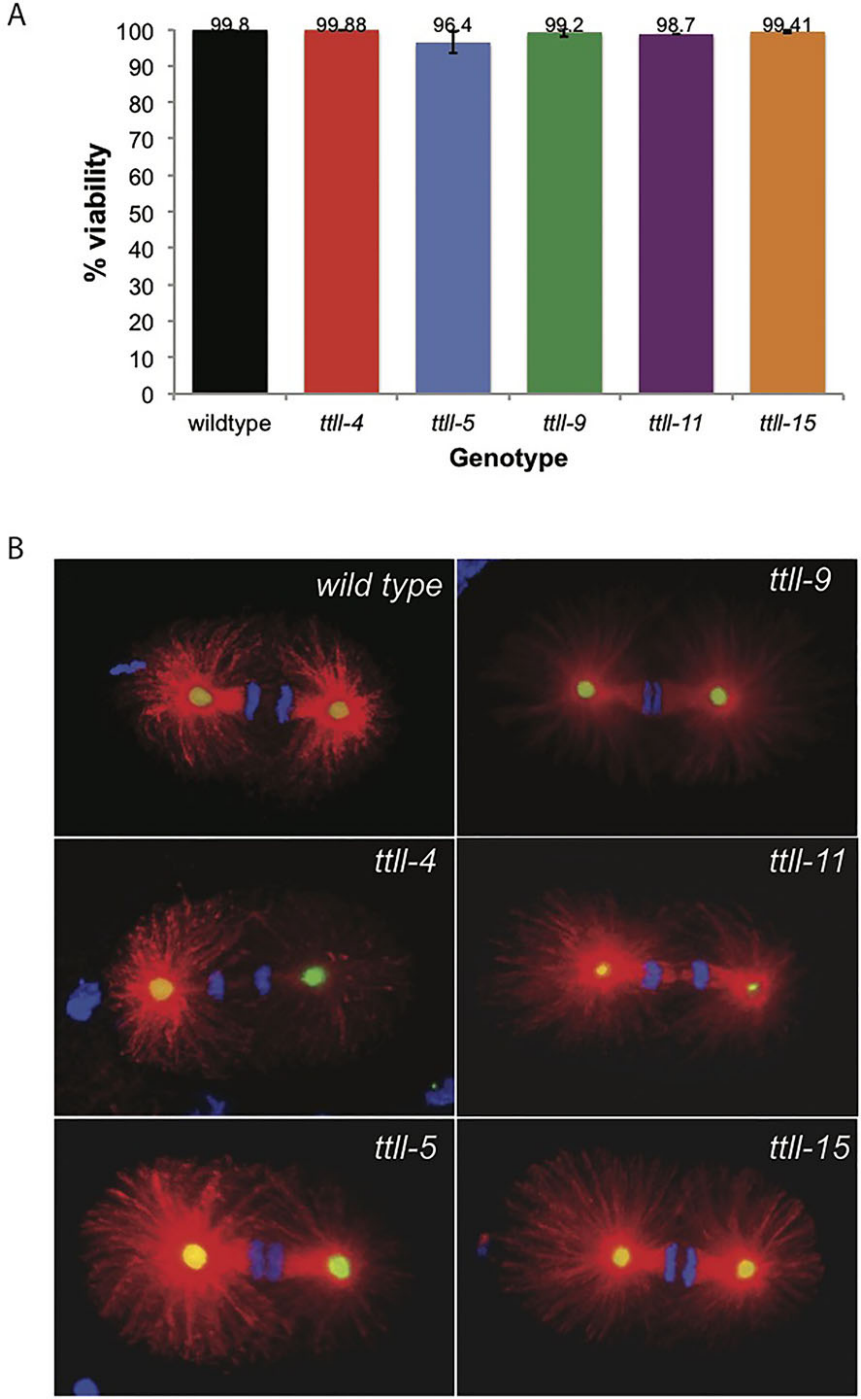
**Individual loss of the TTLL enzymes does not affect embryonic viability or spindle function.** (A) Embryonic viability of all mutants was equivalent to wild type. *n*=15 for each genotype. Error bars indicate s.d. (B) One-cell embryos in anaphase stained for α-tubulin (red), SPD-2 (green) and DNA (blue).

Since all five enzymes are expressed in the embryo, we first wanted to determine whether their activity is necessary for microtubule function in early development. In human cells, tubulin glutamylation is enriched on the microtubules of the centriole and is thought to be essential for centriole stability (Bobinnec et al., 1998a). Early development in *C. elegans* is characterized by rapid cell divisions, and the presence of the centriole is essential for the formation of the spindle and hence for embryonic viability (O'Connell et al., 2001). We therefore assayed embryonic viability as an initial readout for the presence of functional centrioles and spindle microtubules (Fig. 4A). Each mutant showed an embryonic viability close to 100% and was indistinguishable from wild type, which strongly implies that the centrioles are intact and the spindle microtubules are functioning normally. In addition, brood size of the TTLL mutants did not differ from that of wild type, indicating the presence of a fully functional germ line (data not shown).

To directly assess microtubule function in the early *C. elegans* embryo we fixed and stained embryos of each single mutant (Fig. 4B). SPD-2 is a component of both the centriole and pericentriolar matrix (PCM) of the centrosome and all embryos showed SPD-2 staining in one-cell (Fig. 4B) and two-cell embryos (not shown). Furthermore, the centrosomes organized normal bipolar spindles that were able to segregate the chromosomes at anaphase (Fig. 4B). This direct observation, together with a lack of embryonic lethality, confirms that in each TTLL mutant functional centrosomes are present, centrosomes duplicate, and that the microtubules of the spindle are competent for chromosome segregation.

### Normal dye-filling, osmotic avoidance and male mating efficiency suggest cilia are unperturbed in the absence of individual TTLL enzymes

**Fig. 5.**
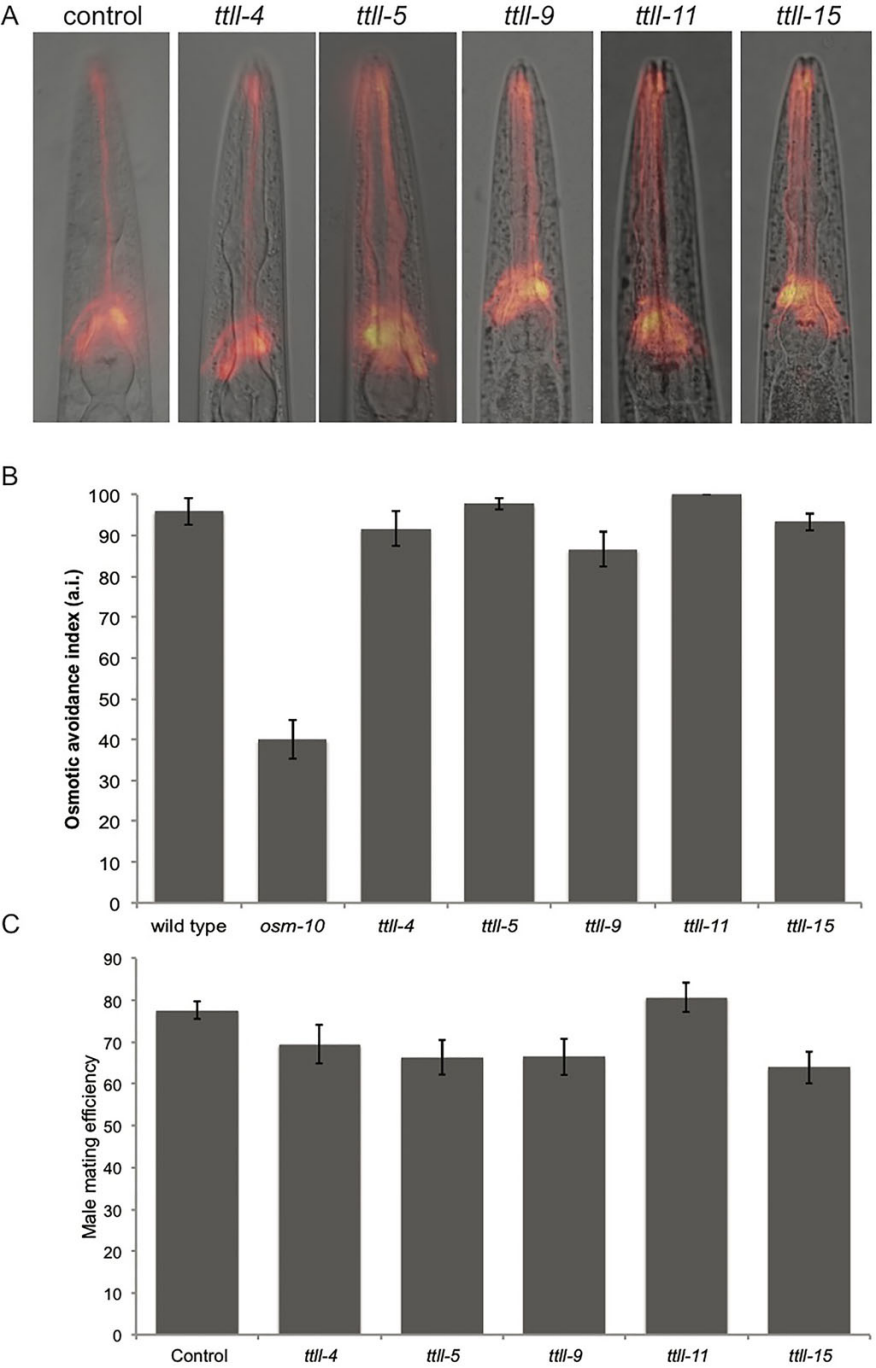
**Dye-filling, osmotic avoidance and male mating efficiency are unaffected in the single mutants.** (A) DiI staining of the amphid neurons (red) is overlaid on a bright field image of the worm. For each strain, 100% of worms showed robust dye-filling (*n*>100). (B) Mean osmotic avoidance of an 8 M glycerol ring. Osmotic avoidance index is the proportion of worms that avoided crossing the ring (for each genotype *n*=90 worms, 5 per trial). *osm-10(n1602)* worms were used as a positive control. Error bars indicate s.e.m. (C) Male mating efficiency is equivalent to wild type. *n*>15 worms for each genotype. Error bars indicate s.e.m.

All five TTLL enzymes are expressed in the adult and we therefore wanted to determine whether they play essential roles in adult processes. Loss of glutamylation has been associated with ciliary dysfunction (Ikegami et al., 2010; Bosch Grau et al., 2013; Kubo et al., 2010; Lee et al., 2013; Suryavanshi et al., 2010) and in *C. elegans*, ciliary microtubules are glutamylated (Bobinnec et al., 1998b; Ikegami et al., 2010; Kimura et al., 2010; O'Hagan et al., 2011), therefore we sought to determine whether cilia function is perturbed in any of the TTLL mutants. In *C. elegans* cilia are confined to sensory neurons, including the amphid neurons in the head and male-specific neurons of the male tail (Inglis et al., 2007). To assay the structural integrity of the amphid cilia we performed dye-filling assays on hermaphrodites (Perkins et al., 1986). Wild-type worms took up the DiI dye through exposed ciliated neuronal tips, and each TTLL mutant also showed normal dye filling (Fig. 5A), indicating that amphid cilia are present. Amphid cilia are required for the worm to avoid noxious chemicals such as those of high osmolarity (Kaplan and Horvitz, 1993), therefore to determine whether function of the amphid cilia was retained we carried out osmotic avoidance assays using *osm-10(n1602)* and wild-type worms as positive and negative controls respectively. All five TTLL mutants showed robust osmotic avoidance of 8 M glycerol (Fig. 5B), similar to wild-type worms, indicating this function of the amphid cilia is retained.

*C. elegans* males possess a greater number of ciliated neurons than hermaphrodites, and glutamylation has been detected on the distal tips of male tail cilia (O'Hagan et al., 2011). When the function of cilia in the male tail is impaired, male mating efficiency is compromised (Barr and Sternberg, 1999). Therefore as an independent assessment of cilia function we assayed male mating efficiency in each TTLL mutant. In order to obtain sufficient numbers of males we combined each TTLL mutation with the *him-9(e1487*) allele which increases non-disjunction in (XX) hermaphrodite meiosis inducing the formation of ∼5% (XO) males (Hodgkin et al., 1979). In each case, the single mutant showed normal male mating efficiency that did not differ significantly from *him-9* controls (Student's *t*-test, *P*>0.05), again suggesting that neuronal and cilia function are unperturbed (Fig. 5C). We therefore find that in the absence of individual glutamylating enzymes the amphid cilia are present and retain chemosensory function, and the male-specific neurons are functional.

### Male mating efficiency is reduced in a triple mutant

**Fig. 6.**
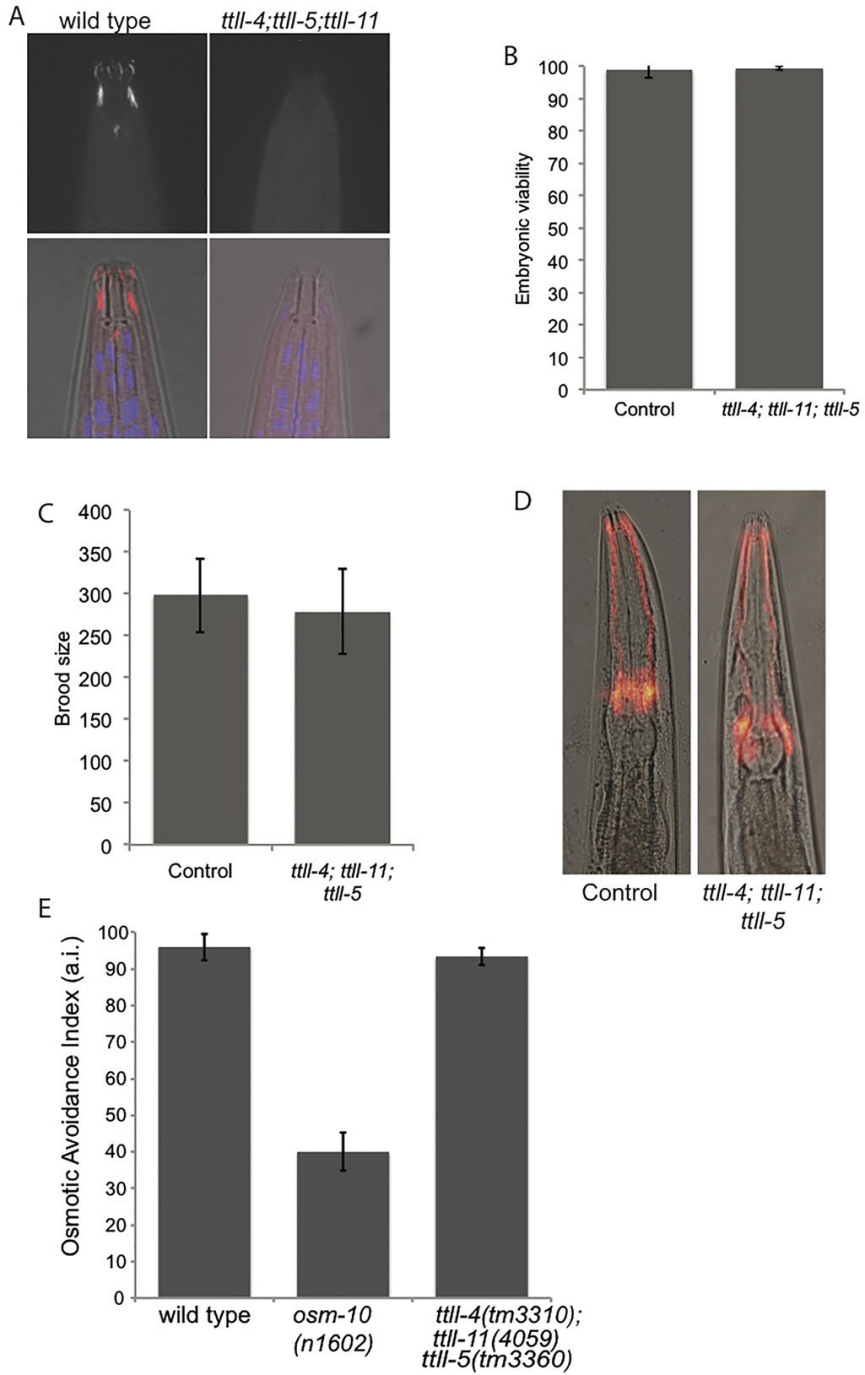
**Viability, brood size, dye-filling and osmotic avoidance are normal in triple mutants.** (A) Glutamylation is undetectable in amphid cilia of *ttll-4(tm3310); ttll-11(tm4059); ttll-5(tm3360)* triple mutants that lack glutamylation initiating enzymes. Embryonic viability (B) and brood size (C) are equivalent to wild type. *n*=15. Error bars indicate s.d. (D) Triple mutants show normal dye-filling indicating that amphid cilia are present. (E) Osmotic avoidance of an 8 M glycerol ring. Osmotic avoidance index is the proportion of worms that avoided crossing the ring (for each genotype *n*=90 worms, 5 per trial). *osm-10(n1602)* worms were used as a negative control. Error bars indicate s.e.m.

**Fig. 7.**
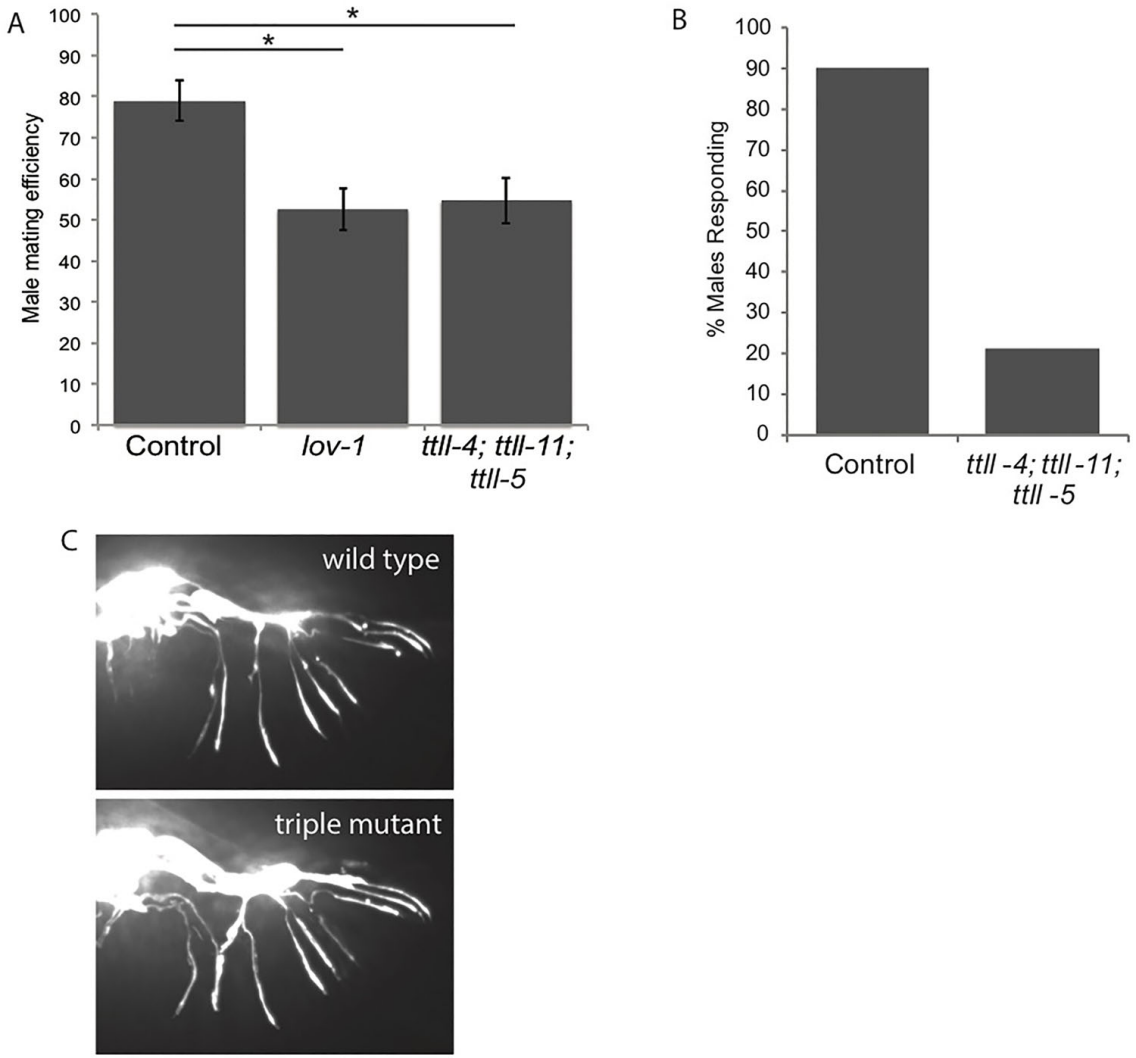
**Triple mutants show male-mating defects.** (A) Male mating efficiency is reduced in the triple mutant. *lov-1(sy552)* was used as a positive control. Error bars indicate s.e.m. *P<0.01, Student's *t*-test. (B) Triple mutant males show a defect in the response step of male mating. Wild type *n*=51 worms; *ttll-4;ttll-11; ttll-5 n*=61 worms. (C) *pkd-2p::GFP* reveals the structure of the male-specific neurons of the male ray in wild type and *ttll-4(tm3310); ttll-11(tm4059);* *ttll-5(tm3360)* triple mutant worms. Small differences in neuron crossing are not significant.

Although we did not uncover defects associated with loss of individual glutamylating enzymes, we reasoned that redundancy in their function may obscure their individual action. By combining individual mutations we were able to test for redundant functions. Since homologs of TTLL-4 and TTLL-5 possess glutamylating-initiating activity (van Dijk et al., 2007), we first combined mutations in these enzymes. The *ttll-4(tm3310); ttll-5(tm3360)* double mutant worms had normal viability and did not show a defect in dye-filling or male mating (data not shown). Although TTLL-11 homologs are largely glutamylation-elongating enzymes, there is evidence that they also possess glutamylation initiating activity (van Dijk et al., 2007), therefore we made a *ttll-4(tm3310); ttll-11(tm4059); ttll-5(tm3360)* mutant (hereafter ‘triple mutant’). We reasoned that this triple mutant would lack all glutamylation-initiating activity, and indeed, we were unable to detect glutamylated tubulin in neurons of the head in triple mutant hermaphrodites (Fig. 6A). Nevertheless, the triple mutant showed normal embryonic viability and brood size (Fig. 6B,C), indicating functionality of microtubules in the embryo and germline. In addition, we observed normal dye-filling and osmotic avoidance, suggesting that the amphid cilia are present and at least partially functional (Fig. 6D). In contrast, we observed significant impairment of male mating efficiency in the triple mutant (Fig. 7A; Student's *t*-test *P*<0.01). Mating efficiency of the triple mutant (55%) was comparable to the negative control *lov-1(sy552)* (53%) which has an established role in male mating due to its function in male-specific cilia (Barr and Sternberg, 1999). Successful male mating involves a stereotyped series of behaviors and to assess which step in this process is impaired in the triple mutant we directly observed male-mating behavior. Upon encountering a hermaphrodite, wild-type males respond by aligning their tail with the hermaphrodite body and begin a backwards motion. When they reach the head or tail, the male will coil around and continue sliding his tail against the hermaphrodite until the vulva is located (Movie 1) (Liu and Sternberg, 1995). In contrast, triple mutant males frequently fail in the initial response step of male mating, failing to align their tail with the hermaphrodite body (Movie 2). Quantification of male mating behavior revealed a substantial defect in the response substep of male mating as >90% of control males responded to hermaphrodites within 5 min (*n*=51), but only 21% of triple mutant males responded within the same time period (*n*=61) (Fig. 7B). Since the response step of male-mating relies on the male-specific ray neurons of the tail (Barrios et al., 2008) we sought to determine whether these neurons were present and structurally intact in the triple mutant. Using a GFP reporter driven by the *pkd-2* promoter, which drives expression in the ray neurons (Schwartz and Horvitz, 2007), we found the neurons of the triple mutant male ray to be indistinguishable from wild type (Fig. 7C). Therefore, the male mating defect we observe in the triple mutant does not stem from loss of the male-specific neurons, instead glutamylation appears to be important for their proper function.

## DISCUSSION

Here we present the first comprehensive *in vivo* analysis of tubulin glutamylating enzymes. In *C. elegans* we find that all five TTLL enzymes are expressed both in the adult and in the embryo. Individual loss of the TTLL enzymes does not impair microtubule function in the centrosome or during cell division. In addition, male mating appears normal and amphid cilia are present and show chemosensory function. However, concurrent loss of three TTLL enzymes leads to reduced male mating efficiency, suggesting these enzymes function redundantly in the male-specific cilia.

Our sequence analysis has revealed conservation of the major features of five TTLL enzymes including the core TTL domain, the extended TTL domain and substrate interaction domains, leading us to conclude that all are glutamylating enzymes. Previous work suggests that loss of TTLL-4 or TTLL-9 reduces the levels of glutamylation in ciliated neurons (Kimura et al., 2010). We confirmed that glutamylation is decreased in the *ttll-4* mutant and additionally find it to be undetectable in *ttll-11* mutants (Fig. 3). In contrast with previous reports, however, we were still able to detect glutamylation in a *ttll-9* mutant, although it is possible that levels of glutamylation may be diminished in comparison to wild type. We speculate that the mutations in *ttll-5* and *ttll-15* do impair enzyme function (the deletions remove essential domains), but that these enzymes do not significantly contribute to glutamylation in the amphid neurons, highlighting likely tissue-specific requirements for the individual enzymes.

Our finding that individual loss of the glutamylating enzymes does not perturb cell division or cilia function was initially surprising, given the evolutionary conservation of the enzymes and roles reported in other species. We suspected that this may be explained by the existence of redundancy between the enzymes for two reasons. First, all five of the TTLL enzymes are expressed in the embryo and adult worm. Second, individual cell types in adult worms express multiple TTLL enzymes (Kimura et al., 2010). Indeed we find that the triple mutant shows reduced male mating efficiency indicating that TTLL-4, -5 and -11 function redundantly. Redundancy between the TTLL enzymes has been observed in other systems; for example depletion of individual TTLL enzymes in mouse ependymal cells does not alter levels of glutamylation, nor did it affect cilia formation or maintenance (Bosch Grau et al., 2013). In trypanosomatids co-depletion of two TTLL enzymes was required to reduce levels of glutamylation (Casanova et al., 2015). Similarly, in *Tetrahymena* co-depletion of *TTLL-1* and *TTLL-9* led to a more severe phenotype than single depletions, again implying redundancy (Wloga et al., 2008). Our data are consistent with the existence of redundancy between the TTLL-4, TTLL-11 and TTLL-5 glutamylating enzymes in the worm. Investigating whether further redundancies exist in *C. elegans* remains an important question since such redundancies could mask additional roles for glutamylation in the worm.

To achieve successful fertilization, *C. elegans* males show a specific series of mating behaviors which rely on the function of a group of male-specific ciliated neurons. Defects in ciliary function manifest as a reduction in male mating efficiency (Liu and Sternberg, 1995). We observe a reduction in male mating efficiency due to a response defect in the triple mutant worms, indicating that the function of these male-specific neurons is impaired. Tubulin glutamylation has been associated with both axonal outgrowth and cilia function, and dysfunction of either could potentially impact male mating behavior. We have confirmed that the male-specific neurons required for male mating behavior are present in the triple mutant worms, which suggests that axonal outgrowth is unaffected. It has previously been shown that hyperglutamylation affects the function of the male-specific cilia (O'Hagan et al., 2011); we therefore speculate that the observed reduction in male-mating efficiency is due to ciliary dysfunction, although we cannot rule out the existence of other subtle perturbations of the neurons such as defects in axon growth and branching. Nevertheless, it appears both increasing and decreasing glutamylation impairs male-specific neuronal function indicating an exquisite sensitivity to glutamylation levels. Intriguingly, our data suggest that the amphid and male-specific neurons are differentially impaired in the triple mutant, implying either that these two cell types have a differential reliance on glutamylation for their function, or that the remaining enzymes supply sufficient glutamylating activity in the amphid neurons. Our inability to detect glutamylated tubulin in amphid neurons of the triple mutants (Fig. 6A) supports the idea that glutamylation is dispensable for amphid cilia function, however it is possible that low levels of glutamylation remain and may suffice for their function.

What role could glutamylation play in the male-specific neurons? PKD-2 is a cation channel that is localized to the cilia in male-specific sensory neurons (Barr et al., 2001). It is required for proper male mating behavior and its loss results in defects in the response substep of male mating and thus reduced male mating efficiency (Barr et al., 2001). A mutation in the deglutamylase CCPP-1 leads to mislocalization of PKD-2 in male-specific CEM cilia suggesting that glutamylation contributes to the proper localization of PKD-2 (O'Hagan et al., 2011). Our triple mutant shows phenotypes reminiscent of loss of PKD-2, which impairs male mating but not amphid ciliary function, therefore we speculate that PKD-2 mislocalization in the *ttll-4; ttll-11; ttll-5* triple mutant underlies the observed male-mating defect. Given that mutation of the PKD-2 homolog *polycystin-2* is associated with polycystic kidney disease in humans, it will be important to test this possibility in the future.

In conclusion, we have characterized the five *C. elegans* glutamylating TTLL enzymes. We find that although evolutionarily conserved, none individually appear to be essential for microtubule function in the worm; however, by combining mutations in three glutamylation-initiating enzymes, we have uncovered a redundant role for three TTLL enzymes in the male-specific neurons.

## MATERIALS AND METHODS

### Worm strains and maintenance

All worms were maintained on MyoB plates seeded with OP50 bacteria. Worm strains used in this study are listed in Table S1. Deletion alleles were obtained from the Japanese Bioresource Center (Gengyo-Ando and Mitani, 2000).

### Protein sequence analysis

Wild-type *C. elegans* gene sequences were obtained in FASTA format from Wormbase (www.wormbase.org). Accession numbers for *C. elegans* protein were: TTLL-4 NP_001022985; TTLL-5 NP_001256331; TTLL-9 NP_001023841; TTLL-11 NP_741471; TTLL-15 NP_505663; TTLL-12 NP_495990*.* Homologous mouse sequences were obtained from the Ensembl database (www.ensembl.org). Accession numbers for mouse protein were: TTLL4 NP_001014974; TTLL5 NP_001074892; TTLL9 NP_001077087; TTLL11 NP_084050; TTLL-12 NP_898838. Using the mouse TTL protein as an out-group, a phylogeny was constructed using the neighbor-joining method with 100 bootstrap replicates to determine closest mouse and worm homologs. TTLL-15 did not resolve with strong bootstrap support, and so a pBLAST search was used to determine its closest mouse homolog. Pairs of homologs were aligned using ClustalX2 and regions homologous to the annotated portions of the mouse protein were identified (Supplementary data) (Garnham et al., 2015; van Dijk et al., 2007). Deletion allele sequences for each *C. elegans ttll* gene were obtained from Wormbase and aligned to their corresponding wild-type alleles. Putative protein sequence for deletion alleles were determined by the ExPASy Translate Tool (web.expasy.org/translate).

### RT-PCR

RNA was extracted from *C. elegans* embryos and adults using TRI Reagent (Sigma) and treated with DNaseI (New England Biolabs). cDNA was made using a superscriptIII first strand synthesis kit (Invitrogen). Primer sequences used for PCR and product sizes were: atgtactcaatttggcag and atctccaattcgttcaag, 242 bp (*ttll-4*); ctgacgagacggagagat and taagacgaaggcgctctc, 192 bp (*ttll-5*); gataaccactgtttcgagctg and ccagtctcaagttaaactgtg, 301 bp (*ttll-9*); ggtcctcaatgctttcag and gagctccataacgagttg, 346 bp (*ttll-11*); aacattttttgatctcag and atgatgccacataacatc, 265 bp (*ttll-15*).

### Embryonic viability and brood size assays

For viability assays, single L4 hermaphrodites were put onto 35 mm plates at 20°C. Each worm was transferred to a new plate every 24 h for three days. Plates were scored after 24 h and the number of viable worms and dead embryos recorded. The embryonic viability from each worm was calculated by dividing the number of viable worms by the total number of offspring laid (Kemp et al., 2007). In all cases, stated viability is the average of *n*>15 worms.

For brood size assays, single L4 hermaphrodites were put onto 35 mm plates at 20°C. Each worm was transferred to a new plate every 24 h until egg laying ceased. The number of progeny, including embryos and worms, on each plate was counted. Total brood size was determined for an individual worm by summing data collected each day (Tissenbaum and Ruvkun, 1998). In all cases stated brood size is the average of *n*>15 worms.

### Imaging

Embryos were fixed and stained using standard protocols (Peel et al., 2012). Primary antibodies were diluted 1:1000: α-tubulin (Dm1A; Sigma); SPD-2 (Kemp et al., 2004). Slides were imaged on a Leica TCS SP8 Using a 100×, 1.25NA objective.

To visualize glutamylation in amphid neurons young adults were washed off plates and fixed in 1× Ruvkin Buffer (80 mM KCl, 20 mM NaCl, 10 mM EGTA, 5 mM spermidine-HCl, 15 mM PIPES, pH 7.4, 25% methanol)+20% formaldehyde, flash frozen in liquid nitrogen and washed in Tris-Triton buffer (100 mM Tris-HCl, pH 7.4, 1% Triton X-100, 1 mM EDTA) (O'Hagan et al., 2011). Worms were incubated in Tris-Triton+1% β-mercaptoethanol overnight and washed in 12 BO_3_ (50 mM H_3_BO_3_, 025 mM NaOH)+0.01% Triton buffer. A 15 min oxidation step in 1× BO_3_+0.01% Triton buffer+0.3% H_2_O_2_ was followed by 1× BO_3_ (50 mM H_3_BO_3_, 025 mM NaOH)+0.01% Triton buffer washes and an antibody buffer B wash (1× PBS, 0.1% BSA, 0.5% Triton X-100, 0.05% sodium azide, 1 mM EDTA). Worms were stored in antibody buffer A (1× PBS, 1% BSA, 0.5% Triton X-100, 0.05% sodium azide, 1 mM EDTA) (O'Hagan et al., 2011). Worms were stained overnight in a 1:600 dilution of GT335 (Adipogen Life Sciences) antibody in antibody buffer A, washed in antibody buffer B and incubated with a 1:2000 dilution of secondary antibody in buffer A. After washing in Antibody buffer B worms were suspended in Vectashield and mounted on 2% agarose pads for viewing using a Diskovery spinning disk confocal system (Andor) mounted on a Nikon Eclipse Ti microscope with a 60×1.4 NA objective.

Recordings of male mating behaviors were made using a SPOT RT3 camera mounted on a Leica MZ16F microscope. To image male tail neurons worms were mounted on an agar pad (8% agarose in M9). They were viewed using a Diskovery spinning disk confocal system (Andor) mounted on a Nikon Eclipse Ti microscope with a 60×1.4 NA objective.

### Dye-filling assay

Worms were incubated in 5 µg/ml DiI (1,1′-dioctadecyl-3,3,3′,3′-tetramethylindocarbocyanine perchlorate) diluted in M9 for 30 min. Subsequently, worms were washed three times in M9 buffer and allowed to crawl on a worm plate for 2 h (Hedgecock et al., 1985). Worms were mounted on an agar pad (8% agarose in M9) and viewed under fluorescent light on a Nikon E800 with a 20×0.75 NA objective.

### Osmotic avoidance behavior assay

Osmotic avoidance was assayed by placing five worms in the center of an 8 M glycerol ring and after 10 min determining the number of worms remaining inside the ring (Hart et al., 1999). The avoidance index was calculated by dividing the number of animals remaining inside the ring by the total number of worms. *osm-10(n1602)* worms were used as a positive control as they are deficient in osmotic avoidance behavior (Hart et al., 1999); N2 worms were used as a negative control. For each genotype *n*=90 worms, five per trial.

### Male mating assays

To assay male mating efficiency, six *unc-52(e444)* L4 hermaphrodites were placed on 35 mm plates with six males. Males and hermaphrodites were allowed to mate for 48 h at 20°C. Males were removed from the plate, and hermaphrodites were transferred to new plates. Hermaphrodites were transferred to new plates every 24 h until egg laying stopped. Plates were scored at 72 h of age. The number of wild type (outcross) and Unc (self) offspring were counted for each plate, and male mating efficiency was calculated by dividing the number of outcross offspring by the total number of offspring (Liu and Sternberg, 1995). Each experimental strain was paired with a *him-9(e1487)* positive control and a *lov-1(sy5522); him5(e1490)* negative control (Barr and Sternberg, 1999). For each genotype *n*=10 trials.

For male mating response assays young males were isolated and kept at 15°C overnight, and then warmed to room temperature before use. Assays were conducted on a plate seeded with 10 µl of concentrated OP50, containing 30 Unc hermaphrodites. Two males were placed in the center of the plate, and individually monitored for the execution of the response step of male mating during a 5 min period. A male was scored as positive if it began scanning a hermaphrodite with his tail and maintained contact for 10 s of more.
